# Verbal autopsy as a tool for identifying children dying of sickle cell disease: a validation study conducted in Kilifi district, Kenya

**DOI:** 10.1186/1741-7015-12-65

**Published:** 2014-04-22

**Authors:** Carolyne Ndila, Evasius Bauni, Vysaul Nyirongo, George Mochamah, Alex Makazi, Patrick Kosgei, Gideon Nyutu, Alex Macharia, Sailoki Kapesa, Peter Byass, Thomas N Williams

**Affiliations:** 1 Kenya Medical Research Institute (KEMRI)/Wellcome Trust Programme, Centre for Geographic Medicine Research-Coast, P.O Box 230, Kilifi, Kenya; 2 INDEPTH Network of Demographic Surveillance Sites, Accra, Ghana; 3 United Nation Statistics Division, New York, USA; 4 Umeå Centre for Global Health Research, Department of Public Health and Clinical Medicine, Umeå University, 90187 Umeå, Sweden; 5 WHO Collaborating Centre for Verbal Autopsy, Umeå University, 90187 Umeå, Sweden; 6 Department of Medicine, Imperial College, London W21NY, UK

**Keywords:** Sickle cell disease, Verbal autopsy, Agreement coefficient, Child mortality, Kenya

## Abstract

**Background:**

Sickle cell disease (SCD) is common in many parts of sub-Saharan Africa (SSA), where it is associated with high early mortality. In the absence of newborn screening, most deaths among children with SCD go unrecognized and unrecorded. As a result, SCD does not receive the attention it deserves as a leading cause of death among children in SSA. In the current study, we explored the potential utility of verbal autopsy (VA) as a tool for attributing underlying cause of death (COD) in children to SCD.

**Methods:**

We used the 2007 WHO Sample Vital Registration with Verbal Autopsy (SAVVY) VA tool to determine COD among child residents of the Kilifi Health and Demographic Surveillance System (KHDSS), Kenya, who died between January 2008 and April 2011. VAs were coded both by physician review (physician coded verbal autopsy, PCVA) using COD categories based on the WHO *International Classification of Diseases* 10^th^ Edition (ICD-10) and by using the InterVA-4 probabilistic model after extracting data according to the 2012 WHO VA standard. Both of these methods were validated against one of two gold standards: hospital ICD-10 physician-assigned COD for children who died in Kilifi District Hospital (KDH) and, where available, laboratory confirmed SCD status for those who died in the community.

**Results:**

Overall, 6% and 5% of deaths were attributed to SCD on the basis of PCVA and the InterVA-4 model, respectively. Of the total deaths, 22% occurred in hospital, where the agreement coefficient (AC_1_) for SCD between PCVA and hospital physician diagnosis was 95.5%, and agreement between InterVA-4 and hospital physician diagnosis was 96.9%. Confirmatory laboratory evidence of SCD status was available for 15% of deaths, in which the AC_1_ against PCVA was 87.5%.

**Conclusions:**

Other recent studies and provisional data from this study, outlining the importance of SCD as a cause of death in children in many parts of the developing world, contributed to the inclusion of specific SCD questions in the 2012 version of the WHO VA instruments, and a specific code for SCD has now been included in the WHO and InterVA-4 COD listings. With these modifications, VA may provide a useful approach to quantifying the contribution of SCD to childhood mortality in rural African communities. Further studies will be needed to evaluate the generalizability of our findings beyond our local context.

## Background

Sickle cell disease (SCD) [1,2] represents a growing health problem both in Africa and in populations of African origin [3]. In a recent study, we estimated that, currently, more than 300,000 children are born with SCD worldwide every year [3]. Approximately three-quarters of these births occur in sub-Saharan Africa (SSA), where facilities for the diagnosis and treatment of SCD are few. As a result, between 50 and 90% of children born with SCD on the continent die undiagnosed in the first 5 years of life [4]. The combination of these high birth rate and mortality figures mean that SCD currently accounts for more than 6% of all deaths among children younger than 5 years in SSA [5].

Poor awareness of SCD among health leaders, combined with a lack of facilities for proper diagnosis and care, means that few data are available regarding such basic questions as the modes and age profile of deaths among children with SCD throughout much of SSA. Recognizing this knowledge gap, we investigated whether verbal autopsy (VA), in which a structured questionnaire is administered to the relatives of deceased people, with a view to ascertaining the probable causes of death, might be one potential approach to investigating the contribution of SCD to childhood deaths in areas where facilities for diagnosis are suboptimal. Here we present data from a study in which we investigated the utility of VA as a tool for diagnosing SCD among children dying in Kilifi County on the coast of Kenya.

## Methods

### Ethics approval

Ethics permission was granted by the KEMRI/National Ethics Review Committee (ERC) in Nairobi and VAs were only administered after obtaining written informed consent from potential respondents.

### Study area

The study was conducted within the area served by the Kilifi Health and Demographic Surveillance System (KHDSS) on the coast of Kenya [6]. Established in October 2000, the KHDSS serves as a framework for population-based epidemiological studies of diseases of local importance, monitors mortality trends, and is used to test and evaluate the impact of public health interventions. The KHDSS covers an area of 891 km^2^ and includes a resident population that currently numbers approximately 260,000. Between 1,200 and 1,500 deaths are recorded each year, of which 240 to 300 are among children older than 28 days and younger than 14 years of age. More than 60% of these deaths occur outside hospital, and their causes are rarely recorded.

Kilifi District Hospital (KDH) provides primary care for the residents of the KHDSS, serving a population of approximately 125,000 children younger than 14 years, and acting as a first-referral hospital for healthcare facilities throughout Kilifi District [7]. The KHDSS forms one component of an integrated health surveillance system: residents of the area are identified at the point of admission to KDH, where their clinical data are immediately entered onto a computerized database, along with the results of a range of routine laboratory investigations [6]. KDH is the only health facility in Kilifi District that provides specialist care to children with SCD.

### Study population and cause of death assignment using verbal autopsy

In collaboration with the Kenyan Ministry of Health (MOH), the KHDSS began collecting VA data in 2008 with the aim of documenting the pattern of underlying cause of death (COD) in the community [8]. The current analysis focuses on residents of the KHDSS area aged 28 days to 14 years who died between January 2008 and April 2011. VAs were administered for these children using the standard 2007 WHO Sample Vital Registration with Verbal Autopsy instrument [8]. VAs were reviewed and coded to provide a maximum of two underlying CODs by two separate methods. First, we used conventional physician coded verbal autopsy (PCVA) in which two independent clinicians reviewed each questionnaire and indicated the underlying COD. As described previously [8], to facilitate comparisons between COD assignment by PCVA and COD assignment by physicians on the hospital wards, the PCVA coding in the current study followed the longer COD list described in the WHO *International Classification of Diseases and Related Health Problems* 10th revision (ICD-10) [9] rather than the more restricted list that is normally used with the WHO 2007 instruments. Importantly, for the purposes of the current study, unlike the restricted WHO 2007 COD list, the ICD-10 list included a code for SCD. Where the two clinicians disagreed or where one could not make a diagnosis, a third clinician was consulted. A COD was assigned when two clinicians agreed on the COD. In instances where there was no agreement between the three clinicians, a consensus COD was reached through arbitration. None of the clinicians had access to data regarding the name or SCD status (affected or unaffected) of deceased and no predetermined diagnostic algorithms were used to attribute CODs to SCD. For the purpose of this analysis, assessments were specifically categorized according to whether or not SCD was mentioned among the underlying CODs. Second, we also used the freely available InterVA-4 computer-based probabilistic model [10] to assign CODs. The 2012 WHO SCD indicator did not map directly to any specific question contained in our questionnaires, which were based on the 2007 WHO instrument. However, these data were partially captured in the free-text sections of the VA forms, for which we developed an automated search for 'sickle OR scd' and mapped it onto the SCD indicator.

### COD assignment in the pediatric ward at KDH

High-quality clinical and laboratory data were available for a subset of children who died in KDH [8]. In order to facilitate comparisons between these different methods of COD assignment, we mapped the ICD-10 COD codes generated by both PCVA and by the hospital physicians to conform to the 2012 WHO VA COD categories that are used by the InterVA-4 software [11] (see Additional file 1).

### Confirmatory laboratory evidence for SCD

Worldwide, a number of genotypes manifest phenotypically as SCD. The principal genotypes include homozygosity for the β^s^ mutation of the *HBB* gene (HbSS), sickle cell-hemoglobin C disease (HbSC) and sickle cell-β-thalassemia. HbSS is the only significant cause of SCD in Kenya [12]. Results of blood tests for HbS typing, conducted by either cellulose acetate hemoglobin electrophoresis (Helena Laboratories, Beaumont, TX, USA) or by high performance liquid chromatography (Variant Analyzer, BioRad, Hercules, CA, USA), were available for a subset of children who were either tested during the course of their admission to KDH or who were members of two prospective cohort studies: (i) the Kilifi Sickle Cell Disease (KSCD) study [13] or the Kilifi Genetic Birth Cohort (KGBC) study [14]. We quantified the contribution of SCD to childhood mortality according to VA, and validated the results in the subset of deaths in children who had been involved in any of these studies, and for whom there was therefore laboratory data that confirmed or refuted a diagnosis of SCD HbSS.

### Data handling

First, we compared data on the underlying CODs recorded by the two VA clinicians to determine inter-reviewer agreement. Second, we compared the underlying CODs reached by PCVA consensus against the InterVA-4 model. Third, for those who had died in KDH, we validated the CODs assigned by PCVA and the InterVA-4.02 model against the COD recorded through the KDH pediatric ward surveillance system. Where more than one COD was given, we selected the underlying COD as our unit of comparison between the VA and pediatric ward COD. Finally, we validated the VA CODs in the subset of deaths with available laboratory data.

### Statistical analysis

We used the agreement coefficient of Gwet (AC_1_) [15,16] to measure levels of agreement (see Additional file 2). Sensitivity and specificity were also calculated to measure diagnostic validity of the VA tool and InterVA-4 model in identifying deaths due to SCD.

Data were transformed using SAS® v.9.2 (SAS Institute Inc., Cary, NC, USA) software and all analyses carried out using R v3.0.0 [17].

## Results

### Total deaths recorded and VAs administered

During the period of the study, a total of 750 deaths among children aged between 28 days and 14 years were recorded through the KHDSS. VAs were administered and PCVA and InterVA-4.02 applied to 610 (81.3%) of these deaths. VAs were omitted for the following reasons: movement of the family from the study area (86; 11.5%), no appropriate respondent identified (30; 4.0%) and refusal of relatives to be interviewed (8; 1.1%). No reason was given for 16 (2.1%). Of those with completed VAs, the median age at death was 2.0 years; 452 (74%) were children aged under 5 years, of whom 262 (58%) were infants. Of the 610 children, 334 (55%) were male. Regarding place of death, 45% of children died in their homes, 43% died in hospital and 12% died elsewhere.

### Agreement between PCVA coders

**Figure 1:**
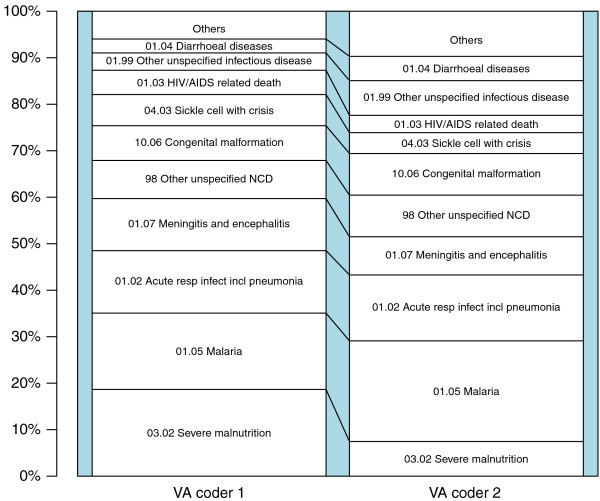
**Cause-specific mortality fractions (CSMFs) for 610 child deaths interpreted by verbal autopsy (VA) coders.** CSMFs for 610 deaths of children aged less than 14 years in the Kilifi Health and Demographic Surveillance System (KHDSS) study area, derived from verbal autopsies interpreted by two independent physician coders.

The cause-specific mortality fractions (CSMFs) assigned by the two primary PCVA coders are summarized in Figure 1 and Additional file 3. The most common diagnoses assigned by both clinicians were malaria, malnutrition, bacterial meningitis, pneumonia, congenital malformations, and SCD. Agreement between the two PCVA coders varied by diagnosis. For example, PCVA coder 1 attributed 84 (14%) and 33 (5%) of the total 610 deaths to malaria and HIV respectively, whereas PCVA coder 2 attributed 96 (16%) deaths to malaria and 21 (3%) to HIV. In addition, PCVA coder 1 attributed 65 (11%) deaths to malnutrition, while PCVA coder 2 attributed only 34 (6%). The overall inter-coder agreement (AC_1_) for all deaths was 54.6% (Table 1), corresponding only to a moderate to high level of agreement [15]. Conversely, however, the AC_1_ for deaths due to SCD was very high [15], at 97.3% (Table 1). Coders did not include SCD in their list of underlying causes of death on the basis of any fixed algorithms, but treated each VA on a case-by-case basis. In some cases, SCD was included under questions 511 and 512, which enquired about pre-existing conditions, in others, the coders appeared to have formed their view on the basis of the free-text elements at the end of the form, which sometimes described characteristic features of SCD, or on the basis of specific responses to questions within the form. Two such questions that appeared to have particular influence were questions 869 and 870, relating to the presence and duration of jaundice, respectively.

**Table 1:** Agreement between the four validation methods in determining the causes of children’s deaths

**Methods**	**AC**_**1 **_**for all CODs, % (SE) (95% CI)**	**AC**_**1 **_**for COD due to SCD, % (SE) (95% CI)**
PCVA coder 1 versus PCVA coder 2 (n = 610)	54.6 (2.0) (50.7 to 58.5)	97.4 (0.6) (95.9 to 98.7)
InterVA-4 model versus PCVA consensus (n = 610)	50.6 (5.0) (48.7 to 57.2)	94.6 (0.9) (95.0 to 99.0)
InterVA-4 model versus paediatric ward COD (n = 134)	42.5 (3.6) (35.4.0 to 49.6)	96.9 (1.5) (95.0 to 99.0)
PCVA consensus versus pediatric ward COD (n = 134)	50.0 (5.1) (40.0 to 60.0)	95.5 (1.8) (92.0 to 99.0)
PCVA consensus versus laboratory evidence (n = 93)	–	87.5 (4.4) (78.9 to 96.1.)

AC_1,_ Agreement coefficient of Gwet; CI, confidence interval; COD, cause of death; SCD, sickle cell disease. SE, Standard error; VA, verbal autopsy.

### Agreement between PCVA consensus and the InterVA-4 model

**Figure 2:**
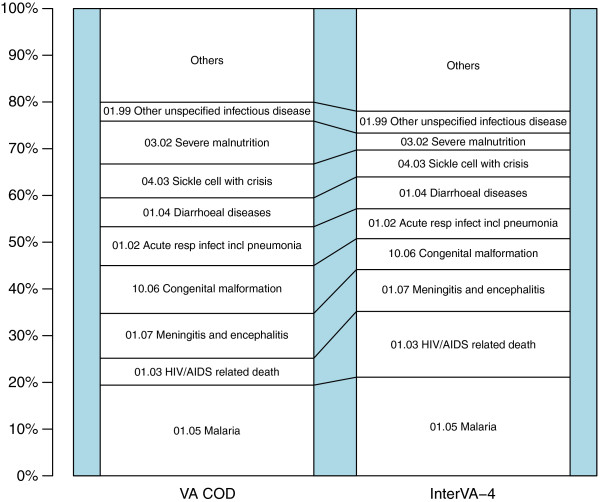
**Cause-specific mortality fractions (CSMFs) for 610 child deaths interpreted by physician coded verbal autopsy (PCVA) and the InterVA-4 model.** CSMFs for 610 deaths of children aged less than 14 years in the Kilifi Health and Demographic Surveillance System (KHDSS) study area, derived from verbal autopsies interpreted by PCVA and the InterVA-4 model.

The CSMFs assigned by PCVA and by the InterVA-4 model are summarized in Figure 2 and Additional file 3. The InterVA-4 model assigned more deaths (13%) to HIV than did PCVA (5%), whereas PCVA attributed 8% of deaths to malnutrition, while the InterVA-4 model attributed only 3%. CSMFs obtained between the InterVA-4 model and PCVA were within ±2% for the other diagnoses, as shown in Figure 2 and Additional file 3. The AC_1_ for all deaths was 50.6%, while the AC_1_ for deaths due to SCD was very high, at 94.6% (Table 1). We noted that the assignment of SCD as the underlying COD both by PCVA and by the InterVA-4 model was strongly influenced by information contained within the free-text section of the questionnaires.

### Agreement between CODs determined by PCVA, the InterVA-4 model, and by clinicians on the KDH pediatric ward

**Figure 3:**
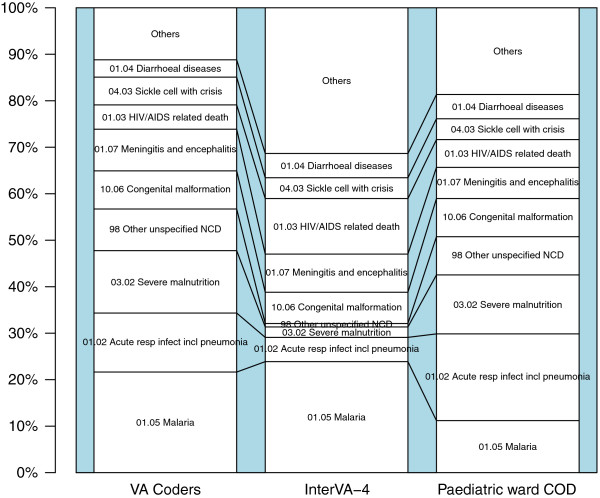
**Cause-specific mortality fractions (CSMFs) for 134 child deaths assigned by physician coded verbal autopsy (PCVA), the InterVA-4 model and pediatric hospital cause of death (COD).** The figure shows CSMFs for 134 child deaths. Underlying CODs determined by PCVA and the InterVA-4 model were compared against the COD given by physicians for those who died on the pediatric ward at Kilifi District Hospital (KDH).

COD was available from the pediatric ward for 134/610 (22%) of children with VA data, for whom we were able to compare data regarding the underlying CODs assigned by the attending clinicians with that assigned both by PCVA and by the InterVA-4 model (Figure 3; see Additional file 3). The CSMFs obtained between PCVA and the InterVA-4 model against the KDH pediatric ward COD were within ±2% for SCD, meningitis, congenital malformation, and diarrheal diseases. PCVA and the InterVA-4 model attributed 13% and 5% of deaths to pneumonia respectively, while 19% of deaths were attributed to pneumonia on the pediatric ward. Similarly, PCVA and the InterVA-4 model assigned almost twice as many deaths to malaria as did clinicians on the pediatric ward. The InterVA-4 model attributed 12% of deaths to HIV/AIDS, whereas the PCVA and the pediatric ward clinicians attributed 5% and 6% of deaths respectively. By contrast, the InterVA-4 model attributed 2% of deaths to malnutrition, while both the PCVA and the pediatric ward clinicians attributed 13%. The InterVA-4 model, PCVA, and pediatric ward clinicians attributed 5%, 6% and 5% of deaths, respectively, to SCD.

### Validation of VA against gold standards

Overall, the AC_1_ between CODs ascribed by PCVA consensus and those assigned by physicians on the pediatric ward was 50.0%, defined as only a moderate to high level of agreement. Similarly, the AC_1_ between the CODs assigned by the InterVA-4 model and those assigned on the pediatric ward was only 42.5% (Table 1). However, the AC_1_ with regard to the specific diagnosis of SCD was 95.5% and 96.9%, respectively, for physician coded VA and the InterVA-4 model (Table 1), consistent with a very high level of agreement. Although physicians on the pediatric ward did not include SCD as one of their underlying CODs on the basis of any fixed diagnostic algorithms, they had the considerable advantage over the PCVA and InterVA-4 methods of being able to take a detailed clinical history, perform a clinical examination, and access specialist tests. In a number of cases, patients were already known attenders at a SCD clinic, and were taking regular prophylaxis for malaria and bacterial diseases. In other cases, patients had a characteristic feature of SCD within their previous medical history, such as a previous admission to hospital in infancy with swollen hands and feet, recurrent admission to hospital with pain in the arms or legs, or frequent admission to hospital with anemia requiring transfusion. In a further group of children, the clinicians’ suspicions appear to have been aroused by findings on clinical examination such as jaundice or splenomegaly, or through observation of the characteristic facial features of SCD. In addition, clinicians had access to data on full blood counts and the ability to confirm their clinical suspicion by blood film examination and electrophoresis. Taking the pediatric ward COD as the gold standard for these reasons, the observed sensitivities and specificities for both the PCVA and InterVA-4 methods across the top 10 major CODs are presented in Table 2. The sensitivity with regard to the diagnosis of SCD was 83% in the case of both PCVA and of VAs interpreted using InterVA-4, while the specificity was 98% and 99%, respectively.

**Table 2:** Validation results for the InterVA-4 model and PCVA consensus against the pediatric ward COD for the top ten CODs among 134 child deaths

**Cause of death**	**Sensitivity, %**		**Specificity, %**		**PPV%**		**NPV%**	
	**InterVA-4**	**PCVA consensus**	**InterVA-4**	**PCVA consensus**	**InterVA-4**	**PCVA consensus**	**InterVA-4**	**PCVA consensus**
1.05: Malaria	80	83	83	90	38	50	97	97
1.02: Pneumonia	14	28	94	91	14	41	81	85
3.02: Severe malnutrition	10	47	97	91	10	44	87	92
98: Other unspecified non-communicable disease	9	18	100	92	100	17	92	93
10.06: Congenital malformation	55	73	98	98	67	73	96	98
1.07: Meningitis	12	44	91	94	15	33	93	96
1.03: HIV/AIDS	40	43	88	95	18	24	94	93
1.04: Diarrheal diseases	29	29	96	98	29	40	96	96
4.03: Sickle cell with crisis	83	83	99	98	83	63	99	99

NPV, negative predicted value; PCVA, physician coded verbal autopsy; PPV, positive predictive value.

### Agreement between PCVA consensus and confirmatory laboratory evidence

To further assess the validity of the VA tool with regard to the identification of deaths attributable to SCD, we also reviewed the COD data determined by VA in the subset of 93 children (15% of 610) for whom laboratory evidence regarding SCD status was available. Of these, 62 (67%) children were phenotype HbAA (normal), 11 (12%) were HbAS (heterozygous carriers) and 20 (21%) were HbSS (that is, they had SCD). The SCD-specific AC_1_ between COD by PCVA and the laboratory phenotype was 87.5% (Table 1). Taking the laboratory data as the gold standard, the sensitivity and specificity of COD according to PCVA for a diagnosis of SCD were 76.9% and 96.6%, respectively.

## Discussion

Although common, SCD remains neglected in many parts of SSA [18]. Few countries have programs for the early diagnosis or specialist treatment of SCD, and the majority of those affected therefore continue to die undiagnosed during early childhood [4]. Although infections, including bacterial diseases [19] and malaria [7,20,21], are widely regarded as probable causes of this early mortality, to a large extent the natural history of SCD in African populations remains poorly documented [18].

In this study, we investigated the question of whether VA might prove a useful approach to the identification of deaths due to SCD in populations for which more robust data on COD are not available. If VA does prove reliable then it could provide a useful tool for improving knowledge on the epidemiology of SCD in resource-poor settings, which could also be helpful both for advocacy and for public health planning.

Consistent with previous studies [22-28], we found that PCVA performed better for some diagnoses than for others. A single previous study was conducted in the same population, which aimed to validate VA against COD data from the hospital wards [25]. In that study, VA was associated with the following sensitivities, specificities and positive predictive values with regard to the attribution of CODs in children older than 1 month, which were broadly similar to those in the current study: acute respiratory infection 28%, 91%, and 29%, respectively; meningitis 38%, 94%, and 20%; and malnutrition 89%, 96%, and 87%. Conversely, the sensitivity of VA for the attribution of malaria was somewhat higher in the current (83%) than in the previous study (46%), although specificity and positive predictive values were similar in both (90% versus 89% and 50% versus 57%, respectively). Measles, a common COD in the earlier study, did not feature among the top 10 diagnoses in the current study, whereas HIV did. Some of these differences might be explained by changes that have been made to the VA tools since the earlier study was conducted, but there have also been significant changes in the patterns of morbidity and mortality in the community in the intervening period. Malaria, previously the single most common cause of admission and death at KDH, has declined significantly in recent years [29], universal childhood vaccination against measles has been introduced, and the HIV epidemic has become well established.

The main focus of the current manuscript, however, is SCD. We found that COD attribution by VA for this diagnosis was consistently good from a number of perspectives. First, agreement between coders was considerably higher for SCD (97.3%) than for all other diagnoses combined (54.6%) (Table 1). Second, agreement between the InterVA-4 model and hospital COD was considerably higher for SCD (96.9%) than for other diagnoses combined (42.5%) (Table 1). Third, agreement between PCVA data and hospital COD was considerably higher for SCD (95.5%) than for other diagnoses combined (50%) (Table 1). Finally, agreement between VA coded data and laboratory-proven SCD was also high at 87.5% (Table 1). Moreover, the proportion of all deaths that were attributed to SCD by VA (6%) was in broad agreement with that predicted from other studies in Africa [5], and is also consistent with that we would predict within the Kilifi population. During the period covered by this study, overall mortality among children aged 1 month to 15 years was in the region of 40/1000, while around 0.8% of all births are affected by SCD [19]. Assuming that 50% of these children die during the first 5 years of life, and that most of these deaths occur after the first month of life [4], SCD should account for around 10% of all deaths among children of this age. Taken together, therefore, these data suggest that the VA method can deliver a plausible estimate of the burden of SCD in our study population.

It is noteworthy that SCD did not feature as a diagnosis either in the previous validation study conducted in Kilifi [25] or in other similar studies [24,27]. The most likely explanation is that no specific code for SCD was included in the shortlist of codes used in these previous VA studies. This is certainly true for the Kilifi study, where deaths from SCD will most likely have been distributed between the classifications 'anemia' and 'others'. Similarly, no specific code for SCD was included even in much larger validation studies such as those published by Setel and colleagues [30] or by Murray and colleagues [31]. Of note, in the latter study, the authors stated that their cause list was constructed based on the WHO Global Burden of Disease estimates of the leading causes of death, potential to identify unique signs and symptoms, and the likely existence of sufficient medical technology to ascertain gold standard cases. This is of particular concern because it reflects the low visibility that SCD currently holds in the eyes of the international health community, despite accounting for rising proportion of declining deaths in children under 5 years of age. In the current study, we used the longer ICD-10 category list for our COD assignment by both PCVA and by hospital physicians, and the WHO 2012 cause list for our COD assignment by InterVA-4. While it is obvious that the absence of a code for SCD in previous code lists will have led to a systematic underestimation of SCD as a COD (to the point of absence) in previous studies, we cannot exclude the possibility that the use of these longer cause lists might have led to an overestimation of SCD as a COD in the current study; further work will be required to investigate this possibility in the future.

During the current study we noted that no specific questions relating to SCD were included in the 2007 WHO VA forms, and that the attribution of SCD as the underlying COD both by PCVA and by the InterVA-4 model therefore leant heavily on the free-text element of the questionnaires. Increasing awareness of the importance of SCD as a major COD in the African context from a number of recent studies [4,5,19,32], and provisional data from this study has now led to the inclusion within the 2012 version of the WHO Verbal Autopsy Instrument of a specific question regarding SCD [11]. Similarly, a specific code for SCD has also now been included in the list of CODs formulated by WHO for VA use, and can also be assigned by the InterVA-4 model. The full process by which this decision, and the decision to introduce or to drop other specific CODs from the 2012 list, were made have been described recently by Leitao and colleagues [33].

Although our study provides new evidence that VA has potential as a tool for identifying deaths due to SCD, it is open to a number of criticisms. Perhaps most importantly, SCD constitutes a major research interest at our program [7,13,19,20] and, as a result, it is possible that our coding clinicians are unusually well versed in the signs and symptoms of SCD. We aimed to mitigate against this bias by applying the InterVA-4 model, which at least guarantees consistency and comparability with possible future studies elsewhere. Similarly, in order to validate our COD assignment by VA, we used as our gold standard COD assignment by a clinician for the subset of deaths that occurred in hospital. We cannot be sure that the distribution of CODs among deaths that occur in hospital is fully representative of CODs overall, and this could potentially lead to an unrecognized and unmeasurable bias. Nevertheless, we suggest that our study would benefit from replication and validation at other sites.

We have previously estimated that approximately 240,000 children are born with SCD annually in Africa alone [3] and that 50-90% of these children die undiagnosed in their first few years of life [4]. Others have estimated that this means that SCD accounts for around 6% of all child deaths in SSA [5], placing SCD firmly on the list of leading causes of death, at least in countries where the gene frequency is high. This is recognized by the welcome inclusion of the hemoglobinopathies in the latest round of the Global Burden of Disease Study [34]. Nevertheless, much more remains to be done to raise the profile of SCD on the global health agenda.

## Conclusion

In summary, we have presented data suggesting that VA might provide a useful approach to investigating the contribution of SCD to childhood deaths in one part of SSA. We hope that our study will prompt similar work in other parts of Africa, including the further exploration of automated approaches to VA coding that might be generalized to other settings.

## Abbreviations

AC1: Agreement coefficient; COD: Cause of Death; CSMF: Cause Specific Mortality Fraction; ERC: Ethics Review Committee; HbSC: Sickle Cell/HbC Disease; HbSS: Sickle Cell Anemia; HIV: Human Immunodeficiency Syndrome; ICD-10: *International Classification of Diseases* 10^th^ Edition; KDH: Kilifi District Hospital; KGBC: Kilifi Genetic Birth Cohort; KHDSS: Kilifi Health and Demographic Surveillance System; KSCD: Kilifi Sickle Cell Disease; MOH: Ministry of Health; PCVA: Physician Coded Verbal Autopsy; SAVVY: Sample Vital Registration with Verbal Autopsy; SCD: Sickle Cell Disease; SSA: Sub-Saharan Africa; VA: Verbal Autopsy; WHO: World Health Organization.

## Competing interests

All authors declare that they have no conflicts of interest.

## Author contributions

CN reviewed the literature, analyzed and interpreted data, and wrote the paper. EB contributed to the study design and helped to edit the final version of the paper. VN helped in interpreting the data and editing of the paper. GM helped to code the VAs, and helped in interpretation of the results and editing of the paper. AMA, PK and SK helped with VA data coding and editing of the paper. GN helped with data management and editing of the paper. AM helped with laboratory data management and editing of the paper. PB helped with interpreting the data and editing the final version of the paper. TNW conceived the study and edited the final version of the paper. All authors read and approved the final version of the manuscript.

## Pre-publication history

The pre-publication history for this paper can be accessed here:

http://www.biomedcentral.com/1741-7015/12/65/prepub

## Supplementary Material

Additional file 1 **Spreadsheet showing mapping of physician coded verbal autopsy (PCVA) and hospital causes of death to the 2012 WHO verbal autopsy (VA) cause of death (COD) categories.** The spreadsheet shows how the PCVA and hospital causes of death were mapped to the 2012 WHO VA categories in order to facilitate comparisons between the three methods. Click here for file

Additional file 2 Calculation of the agreement coefficient of Gwet. Click here for file

Additional file 3 Cause-specific mortality fractions as assigned by the three different methods of cause of death (COD) assignment. Click here for file

## References

[B1] Rees DC Williams TN Gladwin MT Sickle-cell disease Lancet 2010 376 2018 2031 10.1016/S0140-6736(10)61029-X 21131035

[B2] Perutz MF Rosa J Schechter A Therapeutic agents for sickle cell disease Nature 1978 275 369 370 10.1038/275369a0 692720

[B3] Piel FB Patil AP Howes RE Nyangiri OA Gething PW Dewi M Temperley WH Williams TN Weatherall DJ Hay SI Global epidemiology of sickle haemoglobin in neonates: a contemporary geostatistical model-based map and population estimates Lancet 2013 381 142 151 10.1016/S0140-6736(12)61229-X 23103089 PMC3547249

[B4] Grosse SD Odame I Atrash HK Amendah DD Piel FB Williams TN Sickle cell disease in Africa: a neglected cause of early childhood mortality Am J Prev Med 2011 41 S398 S405 10.1016/j.amepre.2011.09.013 22099364 PMC3708126

[B5] Modell B Darlison M Global epidemiology of haemoglobin disorders and derived service indicators Bull World Health Organ 2008 86 480 487 18568278 10.2471/BLT.06.036673 PMC2647473

[B6] Scott JA Bauni E Moisi JC Ojal J Gatakaa H Nyundo C Molyneux CS Kombe F Tsofa B Marsh K Peshu N Williams TN Profile: The Kilifi Health and Demographic Surveillance System (KHDSS) Int J Epidemiol 2012 41 650 657 10.1093/ije/dys062 22544844 PMC3396317

[B7] Komba AN Makani J Sadarangani M Ajala-Agbo T Berkley JA Newton CR Marsh K Williams TN Malaria as a cause of morbidity and mortality in children with homozygous sickle cell disease on the coast of Kenya Clin Infect Dis 2009 49 216 222 10.1086/599834 19514855 PMC2727464

[B8] Bauni E Ndila C Mochamah G Nyutu G Matata L Ondieki C Mambo B Mutinda M Tsofa B Maitha E Etyang A Williams TN Validating physician-certified verbal autopsy and probabilistic modeling (InterVA) approaches to verbal autopsy interpretation using hospital causes of adult deaths Popul Health Metr 2011 9 49 10.1186/1478-7954-9-49 21819603 PMC3160942

[B9] World Health O International Statistical Classification of Diseases and Related Health Problems: ICD-10 1993 Geneva: WHO

[B10] Byass P Chandramohan D Clark SJ D'Ambruoso L Fottrell E Graham WJ Herbst AJ Hodgson A Hounton S Kahn K Krishnan A Leitao J Odhiambo F Sankoh OA Tollman SM Strengthening standardised interpretation of verbal autopsy data: the new InterVA-4 tool Glob Health Action 2012 5 1 8 22944365 10.3402/gha.v5i0.19281 PMC3433652

[B11] Ascertaining and attributing causes of death [http://www.who.int/healthinfo/statistics/verbalautopsystandards/en/]

[B12] Ojwang PJ Ogada T Beris P Hattori Y Lanclos KD Kutlar A Kutlar F Huisman TH Haplotypes and alpha globin gene analyses in sickle cell anaemia patients from Kenya Br J Haematol 1987 65 211 215 10.1111/j.1365-2141.1987.tb02267.x 3828229

[B13] Sadarangani M Makani J Komba AN Ajala-Agbo T Newton CR Marsh K Williams TN An observational study of children with sickle cell disease in Kilifi, Kenya Br J Haematol 2009 146 675 682 10.1111/j.1365-2141.2009.07771.x 19650883 PMC2774158

[B14] Scott JA Berkley JA Mwangi I Ochola L Uyoga S Macharia A Ndila C Lowe BS Mwarumba S Bauni E Marsh K Williams TN Relation between falciparum malaria and bacteraemia in Kenyan children: a population-based, case–control study and a longitudinal study Lancet 2011 378 1316 1323 10.1016/S0140-6736(11)60888-X 21903251 PMC3192903

[B15] Gwet KL Computing inter-rater reliability and its variance in the presence of high agreement Br J Math Stat Psychol 2008 61 29 48 10.1348/000711006X126600 18482474

[B16] Gwet KL Handbook of Inter-Rater Reliability 2 Gaithersburg, USA: Advanced Analytics LLC

[B17] The Comprehensive R Archive Network [http://cran.r-project.org/]

[B18] Makani J Williams TN Marsh K Sickle cell disease in Africa: burden and research priorities Ann Trop Med Parasitol 2007 101 3 14 10.1179/136485907X154638 17244405 PMC5612390

[B19] Williams TN Uyoga S Macharia A Ndila C McAuley CF Opi DH Mwarumba S Makani J Komba A Ndiritu MN Sharif SK Marsh K Berkley JA Scott JAG Bacteraemia in Kenyan children with sickle-cell anaemia: a retrospective cohort and case–control study Lancet 2009 374 1364 1370 10.1016/S0140-6736(09)61374-X 19747721 PMC2768782

[B20] McAuley CF Webb C Makani J Macharia A Uyoga S Opi DH Ndila C Ngatia A Scott JA Marsh K Williams TN High mortality from Plasmodium falciparum malaria in children living with sickle cell anemia on the coast of Kenya Blood 2010 116 1663 1668 10.1182/blood-2010-01-265249 20530796 PMC3073423

[B21] Makani J Komba AN Cox SE Oruo J Mwamtemi K Kitundu J Magesa P Rwezaula S Meda E Mgaya J Pallangyo K Okiro E Muturi D Newton CR Fegan G Marsh K Williams TN Malaria in patients with sickle cell anemia: burden, risk factors, and outcome at the outpatient clinic and during hospitalization Blood 2010 115 215 220 10.1182/blood-2009-07-233528 19901265 PMC2843825

[B22] Snow B Marsh K How useful are verbal autopsies to estimate childhood causes of death Health Policy Planning 1992 7 22 29 10.1093/heapol/7.1.22

[B23] Snow RW Armstrong JR Forster D Winstanley MT Marsh VM Newton CR Waruiru C Mwangi I Winstanley PA Marsh K Childhood deaths in Africa: uses and limitations of verbal autopsies Lancet 1992 340 351 355 10.1016/0140-6736(92)91414-4 1353814

[B24] Mobley CC Boerma JT Titus S Lohrke B Shangula K Black RE Validation study of a verbal autopsy method for causes of childhood mortality in Namibia J Trop Pediatr 1996 42 365 369 10.1093/tropej/42.6.365 9009566

[B25] Quigley MA Armstrong Schellenberg JR Snow RW Algorithms for verbal autopsies: a validation study in Kenyan children Bull World Health Organ 1996 74 147 154 8706229 PMC2486900

[B26] Rodriguez L Reyes H Tome P Ridaura C Flores S Guiscafre H Validation of the verbal autopsy method to ascertain acute respiratory infection as cause of death Indian J Pediatr 1998 65 579 584 10.1007/BF02730899 10773908

[B27] Kahn K Tollman SM Garenne M Gear JS Validation and application of verbal autopsies in a rural area of South Africa Trop Med Int Health 2000 5 824 831 10.1046/j.1365-3156.2000.00638.x 11123832

[B28] Datta N Mand M Kumar V Validation of causes of infant death in the community by verbal autopsy Indian J Pediatr 1988 55 599 604 10.1007/BF02868443 3169930

[B29] O'Meara WP Bejon P Mwangi TW Okiro EA Peshu N Snow RW Newton CR Marsh K Effect of a fall in malaria transmission on morbidity and mortality in Kilifi, Kenya Lancet 2008 372 1555 1562 10.1016/S0140-6736(08)61655-4 18984188 PMC2607008

[B30] Setel PW Rao C Hemed Y Whiting DR Yang G Chandramohan D Alberti KG Lopez AD Core verbal autopsy procedures with comparative validation results from two countries PLoS Med 2006 3 e268 10.1371/journal.pmed.0030268 16942391 PMC1502154

[B31] Murray CJ Lopez AD Black R Ahuja R Ali SM Baqui A Dandona L Dantzer E Das V Dhingra U Dutta A Fawzi W Flaxman AD Gómez S Hernández B Joshi R Kalter H Kumar A Kumar V Lozano R Lucero M Mehta S Neal B Ohno SL Prasad R Praveen D Premji Z Ramírez-Villalobos D Remolador H Riley I Population Health Metrics Research Consortium gold standard verbal autopsy validation study: design, implementation, and development of analysis datasets Popul Health Metr 2011 9 27 10.1186/1478-7954-9-27 21816095 PMC3160920

[B32] Christianson AL Howson CP Modell B March of Dimes Global Report on Birth Defects: The Hidden Toll of Dying and Disabled Children 2006 White Plains, New York: March of Dimes Birth Defects Foundation

[B33] Leitao J Chandramohan D Byass P Jakob R Bundhamcharoen K Choprapawon C de Savigny D Fottrell E Franca E Froen F Gewaifel G Hodgson A Hounton S Kahn K Krishnan A Kumar V Masanja H Nichols E Notzon F Rasooly MH Sankoh O Spiegel P AbouZahr C Amexo M Kebede D Alley WS Marinho F Ali M Loyola E Chikersal J Revising the WHO Verbal Autopsy instrument to facilitate routine cause-of-death monitoring Global Health Action 2013 6 21518 24041439 10.3402/gha.v6i0.21518 PMC3774013

[B34] Murray CJ Vos T Lozano R Naghavi M Flaxman AD Michaud C Ezzati M Shibuya K Salomon JA Abdalla S Aboyans V Abraham J Ackerman I Aggarwal R Ahn SY Ali MK Alvarado M Anderson HR Anderson LM Andrews KG Atkinson C Baddour LM Bahalim AN Barker-Collo S Barrero LH Bartels DH Basanez M-G Baxter A Bell ML Benjamin EJ Disability-adjusted life years (DALYs) for 291 diseases and injuries in 21 regions, 1990–2010: a systematic analysis for the Global Burden of Disease Study 2010 Lancet 2012 380 2197 2223 10.1016/S0140-6736(12)61689-4 23245608

