# Supplementary material for: Verbal autopsy as a tool for identifying children dying of sickle cell disease: a validation study conducted in Kilifi district, Kenya
Source: BMC Med. 2014 Apr 22;12:65. doi: 10.1186/1741-7015-12-65 (PMC4022330; doi:10.1186/1741-7015-12-65)
Supplement: Additional file 2 — Calculation of the agreement coefficient of Gwet. [file 1741-7015-12-65-S2.docx]

**Additional file 2**

***Calculation of the Agreement coefficient of Gwet.***

With two PCVA-coders for a q-level nominal measurement scale (say, q=number of possible types of diseases), we explain our data as shown in Table 1. AC_1_ statistics were calculated as:

$\hat{\gamma}=\frac{p_{a}-p_{e}}{1-p_{e}},$ where

$\hat{\pi}_{k}=(p_{k+}+p_{+k})/2,$ $p_{k+}=n_{k+}/n$ and$p_{+k}=n_{+k}/n$, $p_{a}$is the overall agreement probability and $\hat{\pi}_{k}$ represents the probability of a coder classifying a subject randomly into category *k*. The chance-agreement probability $p_{e}$is a product of two quantities:

1. The probability that 2 raters agree given that the subject being considered was assigned a nondeterministic (random) diagnosis. This conditional probability is $1/q$**.**
2. The probability that a coder will assign a random diagnosis, estimated by:
