# Supplementary material for: Verbal autopsy as a tool for identifying children dying of sickle cell disease: a validation study conducted in Kilifi district, Kenya
Source: BMC Med. 2014 Apr 22;12:65. doi: 10.1186/1741-7015-12-65 (PMC4022330; doi:10.1186/1741-7015-12-65)
Supplement: Additional file 3 — Cause-specific mortality fractions as assigned by the three different methods of cause of death (COD) assignment. [file 1741-7015-12-65-S3.doc]

|  |
| --- |
| **Additional File 3.**  Cause-specific mortality fractions as assigned by the three different methods of COD assignment**.** |

| **Cause of death**† | **CSMFS for all deaths**  **N (%)** | | | | **CSMFs as assigned by the 3 methods for those who died in the hospital**  **N (%)** | | |
| --- | --- | --- | --- | --- | --- | --- | --- |
|  | VA coder 1 | VA coder 2 | PCVA‡ | InterVA-4 | PCVA‡ | InterVA-4 | Paediatric ward |
| **01.02 Acute respiratory infections including pneumonia** | 53 (9) | 50 (8) | 49 (8) | 34 (6) | 17 (13) | 7 (5) | 26 (19) |
| **01.03 HIV/AIDS related death** | 33 (5) | 21 (3) | 30 (5) | 79 (13) | 7 (5) | 16 (12) | 8 (6) |
| **01.04 Diarrhoeal diseases** | 34 (6) | 32 (5) | 31 (5) | 33 (5) | 5 (4) | 7 (5) | 7 (5) |
| **01.05 Malaria** | 84 (14) | 196 (16) | 101 (16) | 108 (18) | 29 (22) | 33 (25) | 15 (11) |
| **01.07 Meningitis and encephalitis** | 53 (9) | 46 (8) | 51 (8) | 53 (8) | 12 (9) | 11 (8) | 9 (7) |
| **01.99 Other unspecified infectious disease** | 16 (3) | 31 (5) | 20 (3) | 24 (4) | 6 (5) | 7 (5) | 3 (2) |
| **03.02 Severe malnutrition** | 65 (11) | 34 (6) | 48 (8) | 18 (3) | 18 (13) | 3 (2) | 17 (13) |
| **04.03 Sickle cell with crisis** | 38 (6) | 30 (5) | 37 (6) | 28 (5) | 8 (6) | 6 (5) | 7 (5) |
| **10.06 Congenital malformation** | 48 (8) | 45 (7) | 51 (8) | 33 (5) | 11 (8) | 9 (7) | 11 (8) |
| **99 Indeterminate** | 21 (3) | 35 (6) | 30 (5) | 80 (13) | 1 (0) | 17 (12) | 0 (0) |
| **Other causes of death** | 166 (27) | 190 (31) | 162 (28) | 120 (20) | 20 (15) | 18 (13) | 31 (24) |
| **Total overall causes** | 610 (100) | 610 (100) | 610 (100) | 610 (100) | 134 (100) | 134 (100) | 134 (100) |

†WHO 2012 VA COD categories; ‡Overall, physician consensus*;* CSMFs are rounded to nearest 1%
